# Sequencing Analysis of *SLX4/FANCP* Gene in Italian Familial Breast Cancer Cases

**DOI:** 10.1371/journal.pone.0031038

**Published:** 2012-02-23

**Authors:** Irene Catucci, Mara Colombo, Paolo Verderio, Loris Bernard, Filomena Ficarazzi, Frederique Mariette, Monica Barile, Bernard Peissel, Elisa Cattaneo, Siranoush Manoukian, Paolo Radice, Paolo Peterlongo

**Affiliations:** 1 IFOM, Fondazione Istituto FIRC di Oncologia Molecolare, Milan, Italy; 2 Unit of Molecular Bases of Genetic Risk and Genetic Testing, Department of Preventive and Predictive Medicine, Fondazione IRCCS Istituto Nazionale dei Tumori, Milan, Italy; 3 Unit of Medical Statistics and Biometry, Fondazione IRCCS Istituto Nazionale dei Tumori, Milan, Italy; 4 Department of Experimental Oncology, Istituto Europeo di Oncologia, Milan, Italy; 5 Division of Cancer Prevention and Genetics, Istituto Europeo di Oncologia, Milan, Italy; 6 Unit of Medical Genetics, Department of Preventive and Predictive Medicine, Fondazione IRCCS Istituto Nazionale dei Tumori, Milan, Italy; 7 Medical Genetics, Department of Medicine, Surgery and Dentistry, Università degli Studi di Milano, Milan, Italy; Ohio State University Medical Center, United States of America

## Abstract

Breast cancer can be caused by germline mutations in several genes that are responsible for different hereditary cancer syndromes. Some of the genes causing the Fanconi anemia (FA) syndrome, such as *BRCA2*, *BRIP1*, *PALB2*, and *RAD51C*, are associated with high or moderate risk of developing breast cancer. Very recently, *SLX4* has been established as a new FA gene raising the question of its implication in breast cancer risk. This study aimed at answering this question sequencing the entire coding region of *SLX4* in 526 familial breast cancer cases from Italy. We found 81 different germline variants and none of these were clearly pathogenic. The statistical power of our sample size allows concluding that in Italy the frequency of carriers of truncating mutations of *SLX4* may not exceed 0.6%. Our results indicate that testing for *SLX4* germline mutations is unlikely to be relevant for the identification of individuals at risk of breast cancer, at least in the Italian population.

## Introduction

Breast cancer is a heterogeneous disease that can be caused by genetic and non-genetic factors. Genetic susceptibility to breast cancer is due to a large spectrum of germline variants. A consistent fraction of familial cases is due to rare high-penetrance mutations in the *BRCA1* and *BRCA2* genes. Other high or moderate penetrance alleles have been identified in genes responsible for hereditary syndromes of which cancer predisposition is one of the features. Namely, these are *TP53* causing the Li-Fraumeni syndrome, *PTEN* causing the Cowden syndrome, *STK11* causing the Peutz-Jeghers syndrome, *CDH1* causing diffuse gastric cancer and *ATM* causing ataxia telangiectasia [1], [2]. Fanconi anemia (FA) is another cancer susceptibility syndrome characterized by genome instability, congenital abnormalities and bone marrow failure. This recessive syndrome is genetically heterogeneous arising from mutations in one of at least 13 different FA genes (FANCA-C, D1, D2, E-G, I, J and L-N). In 2002, the discovery that *BRCA2* and *FANCD1* are the same gene, provided the first evidence of common genetic bases for breast cancer susceptibility and FA [3]. Subsequent studies reinforced this connection showing that truncating mono-allelic mutations in *BRIP1*/*FANCJ* and *PALB2*/*FANCN* had a frequency of approximately 1% in familial breast cancer cases [4], [5]. Moreover, the newly ascertained FA gene *RAD51C* harbored a truncating mutation in 4/1,100 (0.5%) of familial breast cancer cases from Germany [6]. Very recently, bi-allelic truncating mutations of *SLX4* were found in FA patients unlinked to previously identified FA genes [7], [8]. So far, while *BRCA2*, *BRIP1*, *PALB2*, and *RAD51C* are associated with high or moderate breast cancer risk, the impact of *SLX4* on breast cancer remains to be measured. In this context, we sequenced the entire coding region and the intron-exon boundaries of *SLX4* in 526 familial breast cancer cases from Italy.

## Methods

### Patients

The 526 female index cases included in this study were ascertained at the Medical Genetics Units of the Fondazione IRCCS Istituto Nazionale Tumori and the Istituto Europeo di Oncologia in Milan, from March 2003 to August 2008. All subjects, therein referred to as BRCAX cases, were probands of families fulfilling previously reported diagnostic criteria for hereditary breast/ovarian cancer, based on disease family history and age at disease onset [9], [10]. All index cases were verified not to carry deleterious mutations or unclassified variants in *BRCA1* or *BRCA2* genes, following direct sequencing or denaturing high-performance liquid chromatography (DHPLC) analysis of all coding exons and adjacent splice sites, as previously described [9]. All participants in this study signed an informed consent to the use of their biological samples for research purposes. This study was approved by the Ethic Committee of Fondazione IRCCS Istituto Nazionale Tumori, Milan.

### Mutation screening

The mutation analysis was performed by single strand DNA sequencing using primers previously described [7], with the exception of primers for exons 5, 8, 12B-F, 13 and 15 that were re-designed. Sequences of these primers are available upon request.

All newly identified variants have been deposited in the Database of Short Genetic Variations (dbSNP; http://www.ncbi.nlm.nih.gov/projects/SNP/).

## Results and Discussion

In the 526 BRCAX cases, we identified several variants, but no one could be considered as a clear pathogenic mutation. A total of 81 different variants were found and none was truncating (Table S1). Of these variants, 35 were previously annotated in public databases including dbSNP [11], 1000 Genomes [12], and Exome Variant Server (http://evs.gs.washington.edu/EVS/) and reported with carrier frequency ≥1% in Caucasians and thus considered as likely neutral polymorphisms (Table S1). Of the 46 remaining variants, 29 were missense, 14 were silent, two were intronic and one was a 3-nucleotide in-frame deletion causing the loss of a single conserved amino-acid (p.Ile1195del). All 46 variants were analyzed *in silico* to study their potential impact on mRNA splicing using the following four programs: Berkeley Drosophila Genome Project [13], NetGene2 [14], [15], SplicePredictor [16], and GeneSplicer [17], For all but two variants the majority of programs predicted no effect on splicing. The two variants were the c.833G>A, predicted by three programs to create a new donor splice site, and the c.5155T>A, predicted by two programs to abolish the physiological acceptor site of exon 15. These predictions were confirmed using an additional program (MaxEntScan) [18]. The 29 missense variants were studied *in silico* by PolyPhen-2 [19], SIFT [20] and SNP&GO [21] programs and only the p.Ser1719Tyr was classified as a variant with possibly pathogenic effect by all three programs.

Pathogenic mutations of the FA genes *BRIP1*, *PALB2*, and *RAD51C* have been identified in familial cases from different populations with frequencies of 1% to 2% [1], [6], [22]. The vast majority of them are truncating. In Italian breast cancer families, only *PALB2* has been studied and truncating mutations were reported with frequencies ranging between 0.8 and 2.1%[23], [24], [25]. Therefore, the failure of identifying this type of mutations in *SLX4* in a large set of BRCAX cases argues against its role as a breast cancer predisposing gene. In addition, if present, these mutations must be very rare in the Italian population. For our sample size of 526 in which no events (no carriers of truncating *SLX4* mutations) have been observed, the upper limit of the 95% confidence interval of the event probability is 0.006. This indicates that a population frequency of truncating mutations greater than 0.6% would result in a probability of less than 5% of not finding at least one carrier in our sample set and, accordingly, that in the Italian population the frequency of carriers of truncating mutations of *SLX4* may not exceed 0.6%.

We cannot exclude that some of the 81 non-truncating variants we found may be pathogenic or associated with increased risk of breast cancer. In particular, the c.833G>A, c.3583_3585delATT and the c.5155T>A variants were considered to have the higher theoretical probability of being pathogenic, based on *in silico* analyses or phylogenetic conservation. Additional studies, including *in vitro* transcript analyses and functional assays would be required to verify the impact of these mutations on gene functioning. In addition, these variants were very rarely observed in cases (<0,2%) and the assessment of their pathogenicity by case-control studies was not carried out, since it would have required a very large sample size. Segregation analysis was prevented by lack of additional family members. Finally, as another limit of this study, we cannot rule out *SLX4* mutations not detectable by sequence analyses, including copy number alterations and gene rearrangements, could be present in our sample set.

Our data are in line with those from a small study in 52 German and Byelorussian hereditary breast cancer cases, where extensive genetic variation was shown with lack of any clear pathogenic mutation [26], providing evidence against impact of *SLX4* on breast cancer risk in the tested populations. However, *SLX4* might impact on breast cancer risk in populations such as Indians, Americans, and Northern Europeans, where the FA families with *SLX4* truncating mutations originated from [7], [8].

In conclusion, no clear pathogenic mutations were found in 526 Italian BRCAX cases, supporting the hypothesis that *SLX4* is unlikely to be a breast cancer gene. Moreover, our data indicated that truncating mutations of *SLX4* are extremely rare, if not absent, in Italy. Consequently, although our approach might have missed pathogenic *SLX4* alterations, we argue that at present testing for germline mutations in this gene is unlikely to be relevant for the identification of individuals at risk of breast cancer, at least in the Italian population.

## Supporting Information

Table S1 **Variants found in 526 BRCAX cases.**
(DOCX)
