# Supplementary material for: Sequencing Analysis of SLX4/FANCP Gene in Italian Familial Breast Cancer Cases
Source: PLoS One. 2012 Feb 23;7(2):e31038. doi: 10.1371/journal.pone.0031038 (PMC3285620; doi:10.1371/journal.pone.0031038)
Supplement: Table S1 — Variants found in 526 BRCAX cases. (DOCX) [file pone.0031038.s001.docx]

Table S1. Variants found in 526 BRCAX cases

| **Exon** | **Nucleotide change** | **Aminoacid change** | **Mutation type/**  **considered as a neutral polymorphism^a^** | **Annotation status** | **Number of Nor/Het/Hom genotypes; (allelic frequency)** | **Analysis with PolyPhen2/**  **SIFT/SNP&GO** |
| --- | --- | --- | --- | --- | --- | --- |
| 2 | c.60G>A | p.Leu20Leu | silent/no | not annotated | 525/1/0 (0.001) | not done |
| 2 | c.90C>T | p.Ser30Ser | silent/yes | rs118089506 | 511/15/0 (0.014) | not done |
| 2 | c.244A>G | p.Asn82Asp | missense/no | not annotated | 525/1/0 (0.001) | B/T/N |
| 2 | c.247G>A | p.Gly83Ser | missense/no | not annotated | 525/1/0 (0.001) | B/T/N |
| 2 | c.299C>A | p.Thr100Asn | missense/no | not annotated | 525/1/0 (0.001) | B/T/N |
| 2 | c.421G>T | p.Gly141Trp | missense/no | not annotated | 524/2/0 (0.002) | PrD/APF/N |
| 2 | c.452C>T | p.Pro151Leu | missense/no | not annotated | 525/1/0 (0.001) | PoD/APF/N |
| 3 | c.553G>A | p.Asp185Asn | missense/no | not annotated | 525/1/0 (0.001) | B/T/N |
| 3 | c.555C>T | p.Asp185Asp | silent/yes | rs74640850 | 484/42/0 (0.040) | not done |
| 3 | c.590T>C | p.Val197Ala | missense/no | not annotated | 523/3/0 (0.003) | B/T/N |
| 3 | c.610C>T | p.Arg204Cys | missense/yes | rs79842542 | 484/42/0 (0.040) | PrD/APF/N |
| 3 | c.678C>T | p.His226His | silent/yes | rs28516461 | 498/27/1 (0.028) | not done |
| 3 | c.707C>T | p.Ala236Val | missense/no | not annotated | 525/1/0 (0.001) | PoD/T/N |
| 3 | c.710G>A | p.Arg237Gln | missense/no | not annotated | 521/5/0 (0.005) | B/T/N |
| 3 | c.734C>T | p.Pro245Leu | missense/no | not annotated | 524/2/0 (0.002) | B/T/N |
| 3 | c.742G>A | p.Glu248Lys | missense/no | not annotated | 525/1/0 (0.001) | PrD/T/N |
| 3 | c.753G>A | p.Ala251Ala | silent/yes | rs8061528 | 329/172/25 (0.211) | not done |
| 4 | c.761-32T>G | none | intronic/yes | rs118098382 | 514/12/0 (0.011) | not done |
| 4 | c.833G>A | p.Arg278Gln | missense/no | not annotated | 525/1/0 (0.001) | B/T/N |
| 5 | c.999C>T | p.Ile333Ile | silent/yes | rs7198338 | 525/1/0 (0.001) | not done |
| 5 | c.1065G>A | p.Gln355Gln | silent/no | not annotated | 525/1/0 (0.001) | not done |
| 5 | c.1152A>G | p.Pro384Pro | silent/yes | rs112511042 | 484/41/1 (0.041) | not done |
| 5 | c.1153C>A | p.Pro385Thr | missense/yes | rs115694169 | 520/6/0 (0.006) | PoD/T/N |
| 5 | c.1156A>G | p.Met386Val | missense/yes | rs113490934 | 484/41/1 (0.041) | B/T/N |
| 5 | c.1163+10C>T | none | intronic/yes | rs80116508 | 484/41/1 (0.041) | not done |
| 6 | c.1164-75C>G | none | intronic/yes | rs59622164 | 484/42/0 (0.040) | not done |
| 6 | c.1164-66T>A | none | intronic/no | not annotated | 524/2/0 (0.002) | not done |
| 6 | c.1366+11T>C | none | intronic/yes | rs76350200 | 477/48/1 (0.048) | not done |
| 7 | c.1371T>G | p.Asn457Lys | missense/yes | rs74319927 | 488/38/0 (0.036) | B/T/N |
| 7 | c.1641G>A | p.Thr547Thr | silent/no | not annotated | 525/1/0 (0.001) | not done |
| 8 | c.1755C>T | p.Pro585Pro | silent/yes | rs114016359 | 520/6/0 (0.006) | not done |
| 8 | c.1755C>A | p.Pro585Pro | silent/no | not annotated | 525/1/0 (0.001) | not done |
| 8 | c.1803G>A | p.Ser601Ser | silent/no | not annotated | 520/6/0 (0.006) | not done |
| 8 | c.1832C>A | p.Ala611Asp | missense/no | not annotated | 525/1/0 (0.001) | PrD/T/N |
| 8 | c.1846G>A | p.Val616Met | missense/no | not annotated | 525/1/0 (0.001) | PoD/APF/N |
| 8 | c.1896G>C | p.Gly632Gly | silent/no | not annotated | 525/1/0 (0.001) | not done |
| 8 | c.1898G>A | p.Gly633Asp | missense/yes | rs1056085 | 525/1/0 (0.001) | B/T/N |
| 8 | c.1911G>A | p.Ser637Ser | silent/no | not annotated | 525/1/0 (0.001) | not done |
| 9 | c.2006G>A | p.Arg669Asp | missense/no | not annotated | 525/1/0 (0.001) | B/T/N |
| 9 | c.2012T>C | p.Leu671Ser | missense/yes | rs77985244 | 482/44/0 (0.042) | B/T/N |
| 9 | c.2013+23G>A | none | intronic/yes | rs112226642 | 483/43/0 (0.041) | not done |
| 10 | c.2160+50C>T | none | intronic/yes | rs75762935 | 484/42/0 (0.040) | not done |
| 11 | c.2235C>T | p.Thr745Thr | silent/no | rs75184268 | 524/2/0 (0.002) | not done |
| 12 | c.2359G>A | p.Glu787Lys | missense/no | not annotated | 518/8/0 (0.008) | PoD/APF/N |
| 12 | c.2597A>C | p.Gln866Pro | missense/no | not annotated | 525/1/0 (0.001) | PoD/APF/N |
| 12 | c.2854G>A | p.Ala952Thr | missense/yes | rs59939128 | 482/44/0 (0.042) | PrD/T/N |
| 12 | c.2855C>T | p.Ala952Val | missense/yes | rs78637028 | 484/42/0 (0.040) | PrD/T/N |
| 12 | c.2924C>T | p.Pro975Leu | missense/yes | rs114472821 | 519/7/0 (0.007) | B/T/N |
| 12 | c.2975G>A | p.Gly992Glu | missense/no | not annotated | 525/1/0 (0.001) | PoD/T/N |
| 12 | c.3062G>A | p.Arg1021His | missense/no | not annotated | 525/1/0 (0.001) | PoD/T/N |
| 12 | c.3109T>C | p.Leu1037Leu | silent/no | rs58735123 | 525/1/0 (0.001) | not done |
| 12 | c.3162G>A | p.Ser1054Ser | silent/yes | rs76488917 | 500/25/1 (0.026) | not done |
| 12 | c.3189C>T | p.Gly1063Gly | silent/no | not annotated | 511/12/3 (0.017) | not done |
| 12 | c.3308G>A | p.Arg1103His | missense/no | not annotated | 525/1/0 (0.001) | PoD/T/D |
| 12 | c.3316G>A | p.Val1106Met | missense/no | not annotated | 525/1/0 (0.001) | B/T/N |
| 12 | c.3365C>T | p.Pro1122Leu | missense/yes | rs714181 | 462/63/1 (0.062) | B/T/D |
| 12 | c.3420A>G | p.Lys1140Lys | silent/no | not annotated | 525/1/0 (0.001) | not done |
| 12 | c.3583_3585delATT | p.Ile1195del | in frame del/no | not annotated | 525/1/0 (0.001) | not done |
| 12 | c.3662C>T | p.Ala1221Val | missense/yes | [rs3827530](http://www.ncbi.nlm.nih.gov/SNP/snp_ref.cgi?rs=3827530) | 491/35/0 (0.033) | B/T/N |
| 12 | c.3783G>A | p.Pro1261Pro | silent/yes | rs77699867 | 520/6/0 (0.006) | not done |
| 12 | c.3812C>T | p.Ser1271Phe | missense/yes | rs3810813 | 474/52/0 (0.049) | PrD/APF/D |
| 12 | c.3849C>G | p.Ala1283Ala | silent/no | not annotated | 525/1/0 (0.001) | not done |
| 12 | c.3963G>A | p.Pro1321Pro | silent/yes | rs116781836 | 516/10/0 (0.010) | not done |
| 12 | c.4068G>A | p.Pro1356Pro | silent/no | rs115491049 | 525/1/0 (0.001) | not done |
| 12 | c.4338C>T | p.Thr1446Ser | missense/no | rs77718962 | 525/1/0 (0.001) | PrD/T/N |
| 12 | c.4500T>C | p.Asn1500Asn | silent/yes | rs3810812 | 135/246/145 (0.51) | not done |
| 12 | c.4563T>C | p.Pro1521Pro | silent/no | not annotated | 525/1/0 (0.001) | not done |
| 12 | c.4580C>T | p.Pro1527Leu | missense/no | not annotated | 525/1/0 (0.001) | B/T/N |
| 12 | c.4581G>A | p.Pro1527Pro | silent/yes | rs78635099 | 517/9/0 (0.009) | not done |
| 12 | c.4597G>T | p.Ala1533Ser | missense/no | not annotated | 525/1/0 (0.001) | B/APF/N |
| 12 | c.4600G>A | p.Gly1534Ser | missense/no | rs78770603 | 525/1/0 (0.001) | B/T/N |
| 13 | c.4648C>T | p.Arg1550Trp | missense/yes | rs77021998 | 525/1/0 (0.001) | PrD/APF/D |
| 13 | c.4739+24G>T | none | intronic/yes | rs12933120 | 371/143/12 (0.159) | not done |
| 14 | c.4865A>G | p.Gln1622Arg | missense/no | not annotated | 525/1/0 (0.001) | B/T/N |
| 14 | c.5040G>C | p.Arg1680Ser | missense/no | not annotated | 525/1/0 (0.001) | B/T/D |
| 14 | c.5146T>A | p.Ser1716Thr | missense/yes | rs75182789 | 525/1/0 (0.001) | B/T/N |
| 15 | c.5154-28C>T | none | intronic/no | not annotated | 525/1/0 (0.001) | not done |
| 15 | c.5155T>A | p.Ser1719Tyr | missense/no | not annotated | 525/1/0 (0.001) | PrD/APF/D |
| 15 | c.5183T>G | p.Phe1728Cys | missense/no | not annotated | 525/1/0 (0.001) | B/T/D |
| 15 | c.5501A>G | p.Asn1834Ser | missense/yes | rs111738042 | 522/4/0 (0.004) | U/APF/U |
| 15 | c.5505+8A>G | none | intronic/yes | rs3751839 | 476/50/0 (0.048) | not done |

^a^See text for criteria. Abbreviations: Nor, normal; Het, heterozygous; Hom, homozygous. B, benign; PrD, probably damaging; PoD, possibly damaging; U, unclassified (PolyPhen-2). T, tolerated; APF, affecting protein function (SIFT). N, neutral; D, disease; U, unclassified (SNP&GO).
